# Supplementary material for: A Multi-Schedule Machine Learning Pipeline for Medicare Reimbursement Change Prediction and Operational Risk Stratification
Source: medRxiv. 2026 Jul 27:2026.07.22.26358736. Preprint. [Version 1] doi: 10.64898/2026.07.22.26358736 (PMC13484305; doi:10.64898/2026.07.22.26358736)
Supplement: Supplementary file 1 [file NIHPP2026.07.22.26358736v1-supplement-1.pdf]

medRxiv preprint doi: https://doi.org/10.64898/2026.07.22.26358736; this version posted July 27, 2026. The copyright holder for this preprint
(which was not certified by peer review) is the author/funder, who has granted medRxiv a license to display the preprint in perpetuity.
It is made available under a CC-BY 4.0 International license .

Supplementary Material

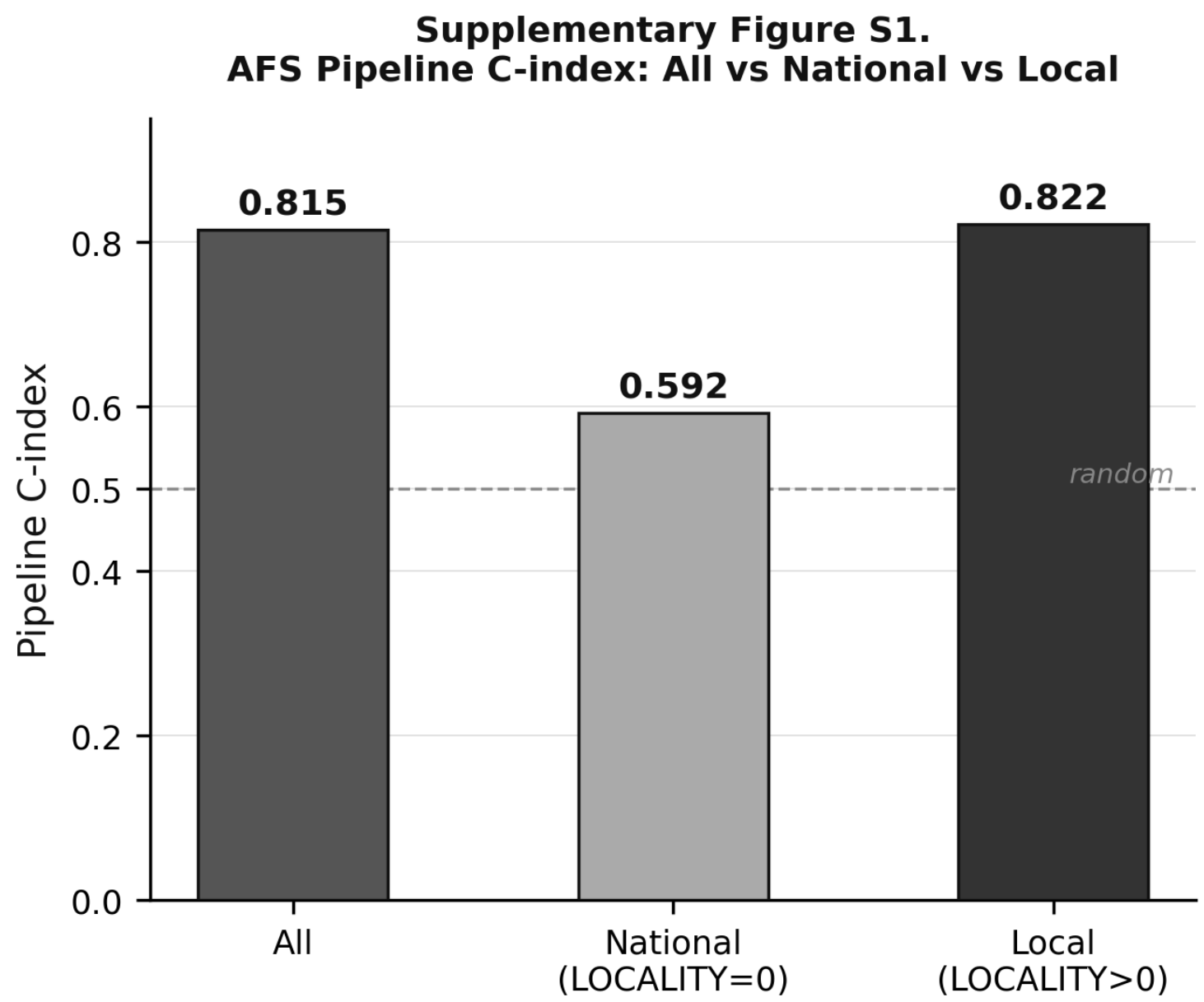

Figure S1: AFS pipeline C-index stratified by locality subgroup. “All” reflects the overall LOGO-CV C-index (0.815). “National” (LOCALITY = 0) reflects performance on codes with no locality adjustment (C-index = 0.592). “Local” (LOCALITY > 0) reflects performance on codes with locality-specific GPCI adjustments (C-index = 0.822). The dashed line indicates chance performance (C-index = 0.5).
